# Bayesian optimal discovery procedure for simultaneous significance testing

**DOI:** 10.1186/1471-2105-10-5

**Published:** 2009-01-06

**Authors:** Jing Cao, Xian-Jin Xie, Song Zhang, Angelique Whitehurst, Michael A White

**Affiliations:** 1 Department of Statistical Science, Southern Methodist University, Dallas, Texas, USA; 2 Department of Clinical Sciences, University of Texas Southwestern Medical Center, Dallas, Texas, USA; 3 Department of Cell Biology, University of Texas Southwestern Medical Center, Dallas, Texas, USA

## Abstract

**Background:**

In high throughput screening, such as differential gene expression screening, drug sensitivity screening, and genome-wide RNAi screening, tens of thousands of tests need to be conducted simultaneously. However, the number of replicate measurements per test is extremely small, rarely exceeding 3. Several current approaches demonstrate that test statistics with shrinking variance estimates have more power over the traditional *t *statistic.

**Results:**

We propose a Bayesian hierarchical model to incorporate the shrinkage concept by introducing a mixture structure on variance components. The estimates from the Bayesian model are utilized in the optimal discovery procedure (ODP) proposed by Storey in 2007, which was shown to have optimal performance in multiple significance tests. We compared the performance of the Bayesian ODP with several competing test statistics.

**Conclusion:**

We have conducted simulation studies with 2 to 6 replicates per gene. We have also included test results from two real datasets. The Bayesian ODP outperforms the other methods in our study, including the original ODP. The advantage of the Bayesian ODP becomes more significant when there are few replicates per test. The improvement over the original ODP is based on the fact that Bayesian model borrows strength across genes in estimating unknown parameters. The proposed approach is efficient in computation due to the conjugate structure of the Bayesian model. The R code (see Additional file 1) to calculate the Bayesian ODP is provided.

## Background

High throughput screening (HTS) is a method for scientific experimentation, which is widely used in drug discovery. It allows researchers to effectively conduct thousands or millions of biochemical or genetic tests simultaneously. Microarray experimentation is a special case of HTS. While one microarray chip can be used to test thousands of genes simultaneously, 96-well plates are typically used in HTS, each well containing one compound. Hundreds or thousands of 96-well plates are needed to test all the compounds once. The number of replicates in HTS is often less than that in microarray experiments, rarely exceeding 3. Nevertheless, they all need to deal with the scenario where the number of statistical comparisons far exceeds the number of biological replicates. To connect with previous methods, we will use "hits" in HTS and "differentially expressed genes" in microarray exchangeably.

Many statistical methods have been developed to identify differentially expressed (DE) genes in microarray experiments. There are variants of Student's *t *test statistic that conduct a test on each individual biological entity and then correct for multiple comparisons. The problem is that, with a large number of tests and a small number of replicates, the statistics are very unstable. For example, a large difference in the measurements under different conditions might be driven by an outlier. Also, a large *t *statistic might arise because of a small variance, even with a small difference in the sample means.

Cui and Churchill [1] used the average of gene-specific variance and pooled variance to estimate variance component. There are several alternative statistics which also modify the estimator of variance. The SAM *t *statistic was proposed in [2] where a suitable constant is added to gene-specific variance estimates. A shrunken *t *statistic [3] was developed with a variance estimator that borrows information across genes using the James-Stein shrinkage idea. In James-Stein estimation, the shrinkage estimate is a linear combination of the original unbiased estimator (sample variance in this case) and a target estimate to minimize a certain loss function (e.g. the mean squared error). This procedure is computationally simple, yet produces efficient estimates. Also in the framework of James-Stein shrinkage, Opgen-Rhein and Strimmer [4] proposed a "shrinkage *t*" approach, which requires no distributional assumption. In general, these analytic shrinkage estimators show a powerful and robust performance in testing DE genes.

From the Bayesian perspective, the introduction of a prior distribution on gene-specific variance naturally implements the shrinkage idea. Baldi and Long [5] proposed the regularized *t *statistic to replace gene-specific variance with a Bayesian estimator based on a hierarchical model. Fox and Dimmic [6] extended Baldi and Long's approach by explicitly calculating the marginal posterior distribution for the difference in mean expression levels. Lonnstedt and Speed [7] proposed an empirical Bayes approach for replicated two-color mi-croarray experiment. Smyth [8] extended the empirical Bayes approach for general microarray experiments. Sartor *et al*. [9] further extended Smyth's method by accounting for the dependence of variance on gene expression intensity. Kendziorski *et al*. [10] considered a hierarchical gamma-gamma model to test DE genes.

Lonnstedt and Britton [11] proposed full Bayesian models and compared them to several highly-used frequentist methods and empirical Bayes methods. They found that the full Bayesian models seem to have less power selecting DE genes. This is because the frequentist test statistics and the empirical Bayes methods, which are similar in performance, put a stronger shrinkage on variance estimates. When the number of replicates is extremely small, the shrinkage becomes more useful in stabilizing the test statistics. In light of this study, we make a simple but important modification by adding a point mass component in the variance prior. It introduces adequate shrinkage in the estimation of variance components so that the full Bayesian model could have equivalent or greater power compared to those highly-used differential expression methods.

The Bayesian model can be combined with frequentist method to further enhance the performance. One of the most current developments in this area is the optimal discovery procedure (ODP) proposed by Storey [12]. Different from the conventional practise of calculating test statistic on each individual gene and then adjusting for multiple comparison, the ODP statistic is calculated based on the information across genes. The method has shown significant gains in power relative to a number of leading methods. To estimate the proportion of the true nulls, Storey used an ad hoc method which is based on ranking the tests by using a univariate statistic (e.g., a *t *statistic). He also used gene-specific sample mean and sample variance to estimate the parameters in the hypothesized null and alternative distributions. In this paper, we propose to use the posterior probability of a gene being DE to estimate the set of true nulls. By doing this, we don't need to choose a cutoff to determine the null set. The uncertainty in the estimation is accounted for in a probabilistic fashion. Furthermore, the sample mean and variance are replaced by the posterior mean and variance of gene expression level. The Bayesian estimates can borrow strength across genes. They may be more reliable than sample mean and variance, which are computed separately for each gene. Our study shows that the Bayesian ODP has considerable improvement over the original ODP, especially when there are few replicates per gene.

## Methods

### The Bayesian model

In this section, we build a full Bayesian hierarchical model, and then we construct the Bayesian ODP statistic to identify DE genes. Let *x*_*ij *_be the expression measurement from the *i*th gene on the *j*th array under the control (*i *= 1,..., *n *and *j *= 1,..., *n*_0*i*_), and *y*_*ik *_be the expression measurement from the *i*th gene on the *k*th array under the treatment (*k *= 1,..., *n*_1*i*_). Replicate number *n*_0*i *_and *n*_1*i *_can be different among genes and between conditions, which means that the Bayesian method can deal with missing values and unbalanced experiment designs. Through a logarithm transformation (or some other transformation) on the original measurements, *x*_*ij *_and *y*_*ik *_are modeled by normal distributions. The first level of the Bayesian model is

$$\begin{array}{lllll} x_{ij}|\mu_{i},\sigma_{i}^{2} & \sim & N(\mu_{i},\sigma_{i}^{2}), & i=1,\cdots ,n, & j=1,\cdots ,n_{0i};\\ y_{ij}|\mu_{i},\Delta_{i},\sigma_{i}^{2} & \sim & N(\mu_{i}+\Delta_{i},\sigma_{i}^{2}), & i=1,\cdots ,n, & k=1,\cdots ,n_{1i}, \end{array}$$

where *μ*_*i *_is the baseline expression level under the control, and Δ_*i *_is the difference in expression levels between treatment and control. We assume that variance $\sigma_{i}^{2}$ is the same under the two conditions for the *i*th gene.

In Bayesian modeling, it is common to introduce a latent variable to indicate the expression status of the *i*th gene [5,7]. Here we use *r*_*i *_= 1/0 to denote differential/nondifferential expression for gene *i*. Specifically, we have

$$\{\begin{array}{ll} \Delta_{i}=0, & \text{if }r_{i}=0,\\ \Delta_{i}\sim \text{N}(0,s_{\Delta }^{2}), & \text{if }r_{i}=1. \end{array}$$

Thus Δ_*i *_is modeled by a mixture of two components, one being a point mass at 0 for non-DE genes, and another being a normal distribution for DE genes. Hyper-parameter $s_{\Delta }^{2}$ is specified as a constant. We further assume that *r*_*i *_| *p*_*r *_~ *Bernoulli*(*p*_*r*_), where *p*_*r *_is the mixing probability.

To introduce a shrinkage on variance component, we impose a mixture structure on $\sigma_{i}^{2}$

$$\{\begin{array}{ll} \sigma_{i}^{2}=\sigma_{0}^{2}, & \text{if }v_{i}=0,\\ \sigma_{i}^{2}\sim \text{IG}(a_{\sigma },b_{\sigma }), & \text{if }v_{i}=1. \end{array}$$

We assume that *v*_*i *_| *p*_*v *_~ *Bernoulli*(*p*_*v*_), where *p*_*v *_serves as the mixing probability. Thus *v*_*i *_= 0 indicates that gene *i *shares a common variance with some other genes, and *v*_*i *_= 1 indicates that it has a gene-specific variance arising from a continuous inverse gamma distribution. We specify hyper-parameters *a*_*σ *_and *b*_*σ *_as constants.

We complete the Bayesian model with prior specifications for parameters (*μ*_*i*_, $\sigma_{0}^{2}$, *p*_*r*_,*p*_*v*_),

$$\begin{array}{ccc} \mu_{i} & \sim & N(0,s_{\mu }^{2}),\\ \sigma_{0}^{2} & \sim & IG(a_{0},b_{0}),\\ p_{r} & \sim & Beta(a_{r},b_{r}),\\ p_{v} & \sim & Beta(a_{v},b_{v}), \end{array}$$

where ($s_{\mu }^{2}$, *a*_0_, *b*_0_, *a*_*r*_, *b*_*r*_, *a*_*v*_, *b*_*v*_) are specified as constants.

Let *X *and *Y *be the collections of expression measurements from all the genes under control and treatment, respectively. Our primary interest is *z*_*i *_= *E*(*r*_*i *_| *X*, *Y*), the marginal posterior probability that gene *i *is DE. We use *z*_*i *_as the test statistic, i.e., a gene is flagged as DE if *z*_*i *_> *λ*, where *λ *is a cutoff value.

Computing *z*_*i *_involves integration over all the other parameters in the joint posterior distribution. This integration does not have a closed form. We implement a Markov Chain Monte Carlo (MCMC) algorithm to make posterior inference. All the full conditional distributions are of standard forms such as normal, inverse gamma, beta, and Bernoulli distributions, so it is efficient to run the MCMC simulation.

### The Bayesian ODP

Multiple testing methods are typically based on *p*-values obtained from each hypothesis test, which only uses information from individual tests. Because there is often a strong biological structure among HTS tests, the measurements from different tests can be related. Storey [12] proposed the optimal discovery procedure (ODP) to construct a test statistic using information across tests. Denote the expected number of true positives as ETP and the expected number of false positives as EFP. The ODP is optimal in that it maximizes the ETP for each fixed EFP level. The method has shown significant gains in power relative to a number of current leading methods.

Here is the outline of the ODP. Suppose there are *n *tests, and test *i *has null density *f*_*i *_and alternative density *g*_*i*_, for *i *= 1,..., *n*. The observed data are **x**_1_, **x**_2_,..., **x**_**n**_, where **x**_**i **_corresponds to test *i*. Then the ODP test statistic is

$$S_{ODP}(x)=\frac{\begin{array}{c} \text{sum of probability of data }x\\ \text{under each true alternative distribution} \end{array}}{\begin{array}{c} \text{sum of probability of data }x\\ \text{under each true null distribution} \end{array}}.$$

Because the true parameters in the null and alternative distributions are unknown, Storey *et al*. [13] proposed the canonical plug-in estimate

(1) $${\hat{S}}_{ODP}(x)=\frac{\sum_{i=1}^{n}{\hat{g}}_{i}(x)}{\sum_{i=1}^{n}{\hat{w}}_{i}{\hat{f}}_{i}(x)},$$

where ${\hat{f}}_{i}$ and ${\hat{g}}_{i}$ are the estimates of *f*_*i *_and *g*_*i*_, ${\hat{w}}_{i}$ = 1 if ${\hat{f}}_{i}$ is to be included in the denominator, and ${\hat{w}}_{i}$ = 0 otherwise. Specifically, the authors [13] assumed that the expression measurements follow a normal distribution, and they proposed to plug in the constrained maximum likelihood estimates under *f*_*i *_and the unconstrained maximum likelihood estimates under *g*_*i*_. The estimates are the sample mean and sample variance under the hypothesized normal distribution. To estimate the null set, Storey *et al*. suggested an ad hoc approach to estimate *w*_*i*_. First, rank the tests using a univariate statistic (e.g., *t *statistic). Second, decide a cutoff, and the tests with the univariate statistic falling below the cutoff are classified into the null set (${\hat{w}}_{i}$ = 1). The cutoff is chosen where the proportion of statistics not exceeding the cutoff equals the estimated proportion of true nulls based on the method in [14]. Finally, a null hypothesis is rejected if ${\hat{S}}_{ODP}$(**x**_**i**_) exceeds some cutoff chosen to attain a given EFP level.

The above ad hoc approach can be improved because the distributional parameters are estimated only based on information from individual tests. The posterior estimates from the proposed Bayesian model allow borrowing strength across all tests, which could provide more stable estimates. We propose to use the posterior means of *μ*_*i*_, *μ*_*i *_+ Δ_*i*_, and $\sigma_{i}^{2}$ to estimate the parameters of *f*_*i *_and *g*_*i *_in the ODP statistic.

One way to estimate *w*_*i *_is to decide a cutoff on the posterior probability (*z*_*i*_) of a gene being DE, i.e., ${\hat{w}}_{i}$ = 0 if *z*_*i *_is greater than the cutoff (e.g., 0.5) and ${\hat{w}}_{i}$ = 1 otherwise. Storey *et al*. [13] suggested that *w*_*i *_can be thought of as weights estimating the true status of each hypothesis, and they could take on a continuum of values. Then another option is to set ${\hat{w}}_{i}$ = 1 - *z*_*i*_, the probability of the *i*th gene being non-DE, which can also be interpreted as the probability of the *i*th null hypothesis being true. The natural introduction of the posterior probability into the ODP statistic overcomes the problem of choosing an arbitrary cutoff value. It also accommodates the uncertainty in estimating the true status of each test. In this paper, we implement this second option to construct the Bayesian ODP statistic.

## Results and discussion

We conducted simulation studies and data analysis based on two experimental datasets to assess the performance of the Bayesian ODP. It is compared to six methods in identifying DE genes: the original ODP, the posterior probability from the Bayesian mixture model, the shrunken *t *[3], Fox and Dimmic's Bayesian *t *(Fox) [6], the moderated *t *[8], and the intensity-based moderated *t *(IBMT) [9].

### Simulation study

**Figure 1:**
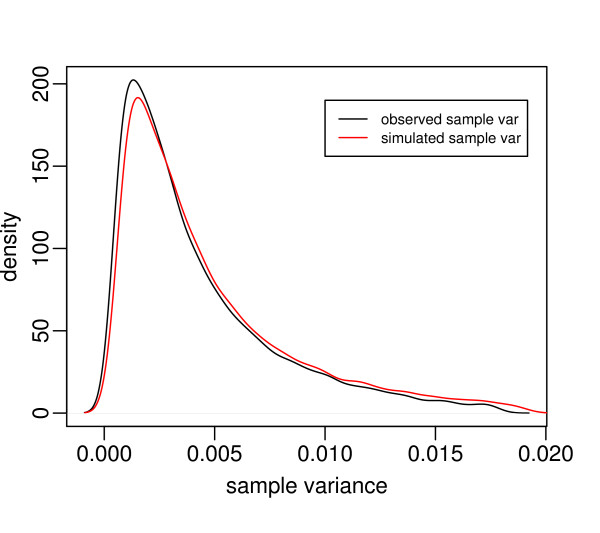
**The empirical density curves of the observed sample variances and the simulated sample variances for the lung cancer data, respectively**.

We simulated data based on the estimated parameters from the HTS lung cancer data set described next. Specifically, we used an inverse gamma distribution to model the gene variance components. Figure 1 plots the empirical density curves of the observed sample variances and simulated sample variances based on the inverse gamma model. The two curves are similar, except that the curve based on the observed sample variances is relatively more spiked in the center. The difference can be accommodated by assuming that some genes have a common variance around the mean of the gene-specific variances. In the simulation, we used the inverse gamma model to generate gene-specific variances $\sigma_{i}^{2}$,

$$\{\begin{array}{ll} \sigma_{i}^{2}=\sigma_{0}^{2}, & \text{if }v_{i}=0,\\ \sigma_{i}^{2}\sim \text{IG}(2.3,0.01), & \text{if }v_{i}=1, \end{array}$$

where we set the common variance $\sigma_{0}^{2}$ to be the mean of the gene-specific variances. Without loss of generality, we assumed that the mean expression level under control equals 0 (*μ*_*i *_= 0). The difference in expression levels between treatment and control is specified as

$$\{\begin{array}{ll} \Delta_{i}=0, & \text{if }r_{i}=0,\\ \Delta_{i}\sim \text{N}(0,0.12), & \text{if }r_{i}=1. \end{array}$$

We conducted simulation studies with 2 to 6 replicates per gene. We considered two scenarios for a given number of replicates. In Scenario 1, all gene variances are gene-specific; in Scenario 2, 80% of gene variances are gene-specific and 20% of genes have a common variance. One hundred datasets were simulated under each scenario, where each dataset contains 1000 genes with 100 genes being DE.

We used noninformative priors so that posterior inference is dominated by the information from data. Specifically, we let $s_{\mu }^{2}$ = ${\hat{S}}_{ODP}$ = 1.0 where 1.0 is sufficiently large for the expression levels. To specify the hyper-parameters for the inverse gamma priors, first we set *a*_*σ *_= *a*_0 _= 2.0 so that the inverse gamma priors have an infinite variance. Then we let the prior means, $\frac{b_{\sigma }}{a_{\sigma }-1}$ and $\frac{b_{0}}{a_{0}-1}$, equal to the average of the sample variances to solve for *b*_*σ *_and *b*_0_. Finally, we choose *a*_*r *_= *b*_*r *_= *a*_*v *_= *b*_*v *_= 1, which corresponds to the uniform priors for *p*_*r *_and *p*_*v*_. The computation is done by Gibbs sampling with 11,000 cycles. The burn-in is 1,000. We monitor two parallel chains with different starting points to assess convergence.

**Figure 2:**
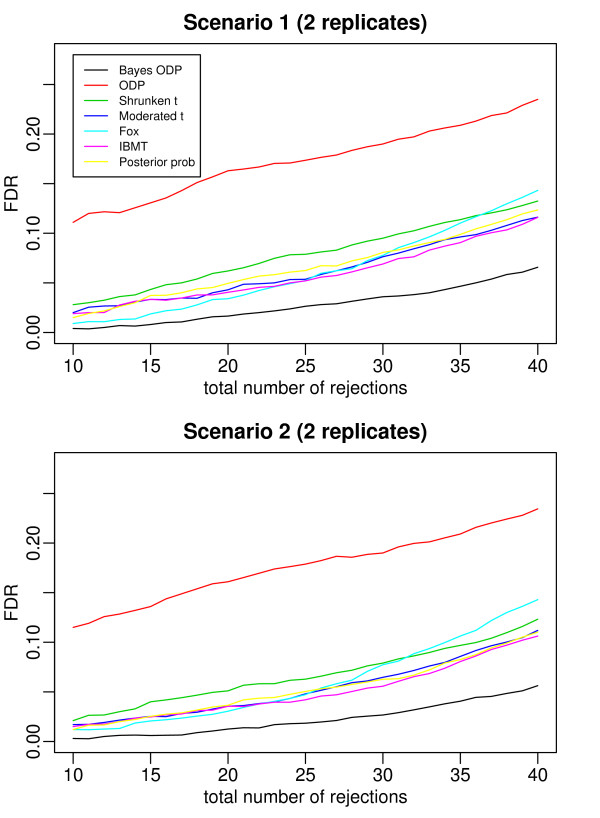
**ROC curves which compare the Bayesian ODP, the original ODP, the posterior probability from the Bayesian model, the shrunken *t*, the moderated *t*, Fox, and IBMT**. The number of replicates per gene is 2. In Scenario 1, gene variances are gene-specific; in Scenario 2, 80% of gene variances are gene-specific and 20% of genes have a common variance.

**Figure 3:**
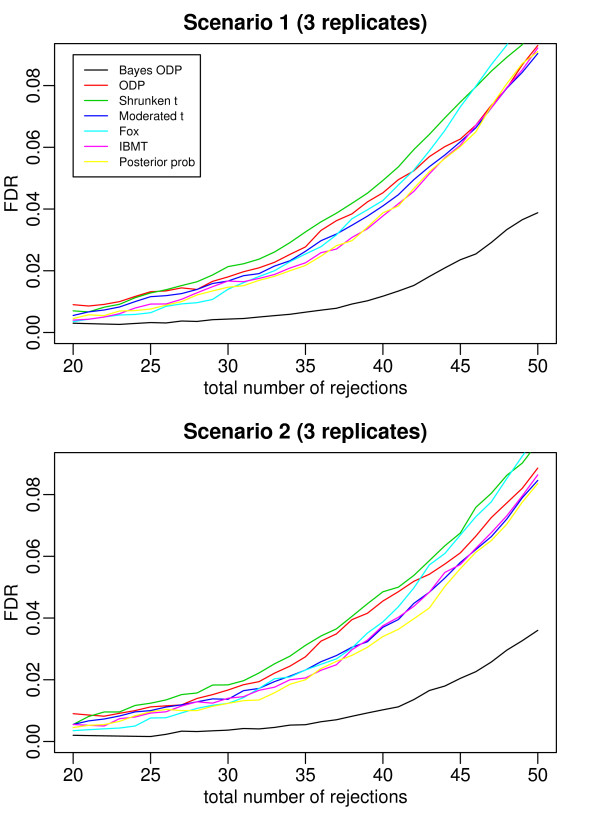
**ROC curves which compare the Bayesian ODP, the original ODP, the posterior probability from the Bayesian model, the shrunken *t*, the moderated *t*, Fox, and IBMT**. The number of replicates per gene is 3. In Scenario 1, gene variances are gene-specific; in Scenario 2, 80% of gene variances are gene-specific and 20% of genes have a common variance.

**Figure 4:**
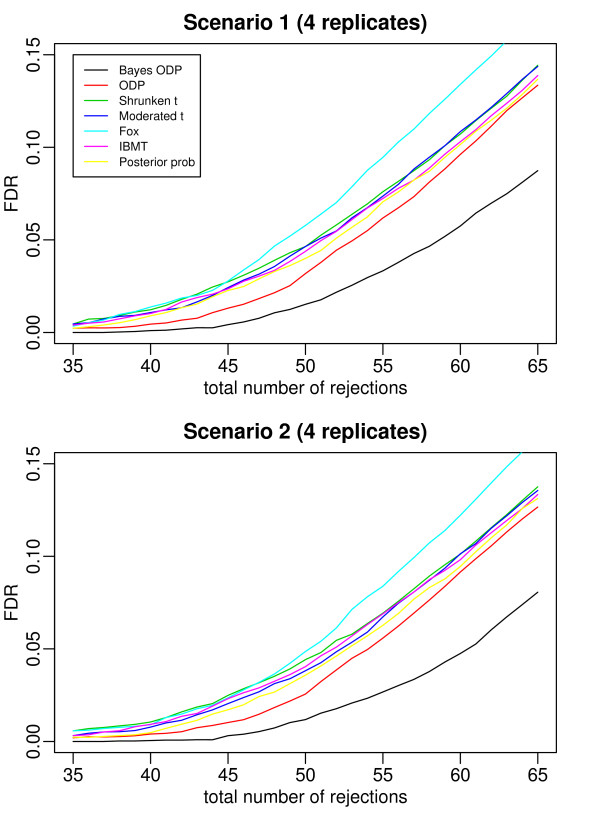
**ROC curves which compare the Bayesian ODP, the original ODP, the posterior probability from the Bayesian model, the shrunken *t*, the moderated *t*, Fox, and IBMT**. The number of replicates per gene is 4. In Scenario 1, gene variances are gene-specific; in Scenario 2, 80% of gene variances are gene-specific and 20% of genes have a common variance.

**Figure 5:**
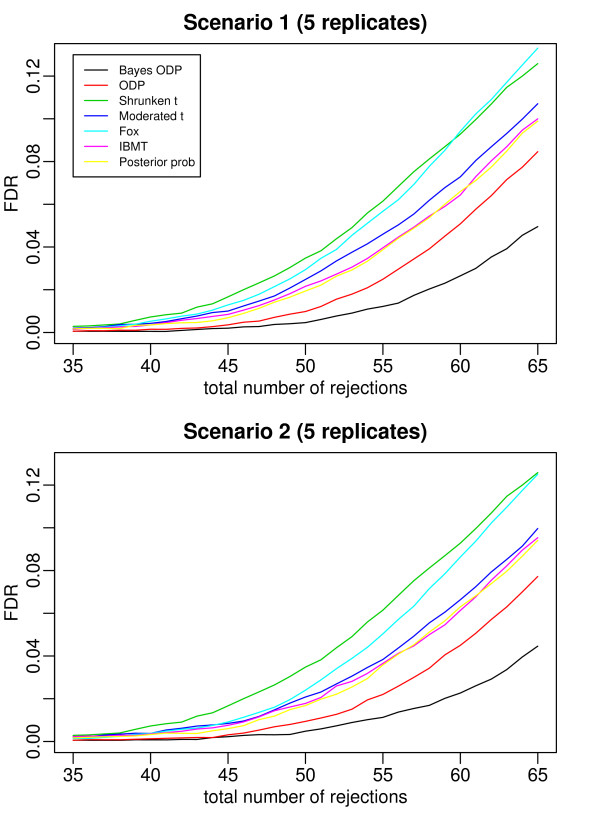
**ROC curves which compare the Bayesian ODP, the original ODP, the posterior probability from the Bayesian model, the shrunken *t*, the moderated *t*, Fox, and IBMT**. The number of replicates per gene is 5. In Scenario 1, gene variances are gene-specific; in Scenario 2, 80% of gene variances are gene-specific and 20% of genes have a common variance.

**Figure 6:**
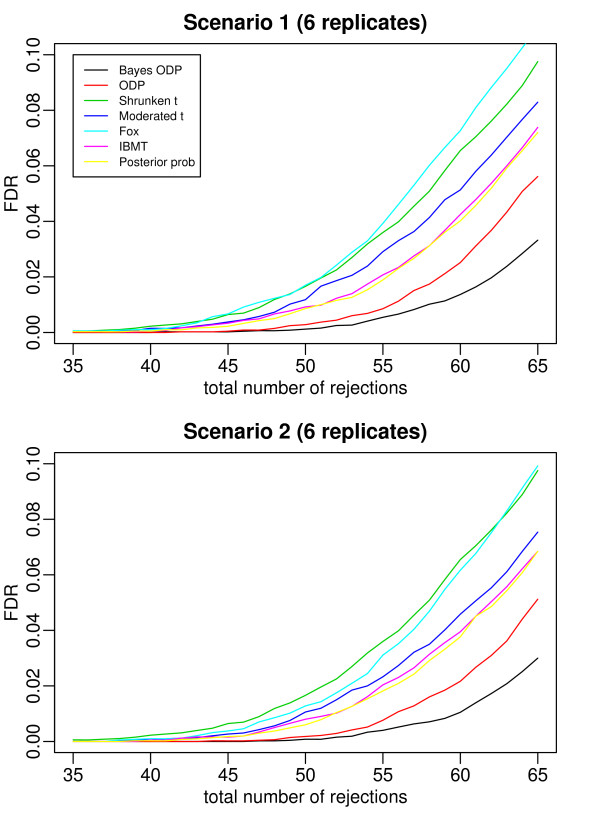
**ROC curves which compare the Bayesian ODP, the original ODP, the posterior probability from the Bayesian model, the shrunken *t*, the moderated *t*, Fox, and IBMT**. The number of replicates per gene is 6. In Scenario 1, gene variances are gene-specific; in Scenario 2, 80% of gene variances are gene-specific and 20% of genes have a common variance.

Figures 2, 3, 4, 5, and 6 plot the false discovery rate (FDR) versus the number of rejected genes with 2 to 6 replicates per gene. The top panel is under Scenario 1 and the bottom panel is under Scenario 2. In general, the two plots in each figure show a similar pattern, indicating that the true percentage of genes having a common variance does not affect the results much. The introduction of the mixture model on variance components is useful even when all the variance components are gene-specific. In all the cases considered, the Bayesian ODP significantly outperforms the others, including the original ODP. The posterior probability shows similar performance as the shrunken *t*, the moderated *t*, Fox, and IBMT. The extra shrinkage introduced by the mixture distribution on variance components makes the full Bayesian model comparable to the shrinkage and empirical Bayes statistics.

In [13], the ODP shows significant improvement over the shrunken *t *statistic. However, in our simulation study, the ODP has the worst performance with 2 replicates per gene. It performs comparably to the shrunken *t *with 3 or 4 replicates per gene, and it outperforms the shrunken *t *with 5 or 6 replicates. The reason might be that, in [13] each gene was tested on a relatively large number of arrays, i.e., with six, seven, and eight replicates under three conditions, respectively. The sample mean and sample variance, which are used in the ODP statistic defined in (1), are much more stable compared to those based on few replicates. As shown in [3], the fewer replicates there are, the more the shrinkage is introduced in the shrunken *t *statistic. In such cases, the ODP, which uses sample mean and variance, might be outperformed by the shrinkage method. As the number of replicates increases, sample variance becomes more stable, the benefit of the shrinkage becomes less significant, and the advantage of the ODP statistic can be revealed.

The Bayesian ODP is constructed based on the ODP test statistic, which has been shown to have optimal performance in multiple significance tests [12]. It also takes advantage of the parameter estimates from the Bayesian mixture model which are more reliable than those in the original ODP. When the number of replicates is extremely small, the Bayesian ODP might have a better performance in identifying DE genes.

### Experimental datasets

In this section, we applied the Bayesian ODP to two experimental datasets. The first dataset is from a real HTS experiment. Paclitaxel and related taxanes are routinely used in the treatment of non-small cell lung cancer and other epithelial malignancies. The goal of the experiment is to identify gene targets that specifically reduce cell viability in the presence of paclitaxel. Whitehurst *et al*. [15] designed an HTS experiment which combined a high throughput cell-based one-well/one-gene screening platform with an arrayed genome-wide synthetic siRNA library for systematic interrogation of the molecular underpinnings of cancer cell chemoresponsiveness. The information on the dataset can be accessed from the *Nature *website . The dataset was generated under two conditions (in the presence and absence of paclitaxel). Over 21,000 genes were measured, each with 3 replicates. The measurements are the cell viability scores based on Adenosine TriPhosphate (ATP) concentration.

The raw data were normalized to internal reference control samples on each plate to allow for plate-to-plate comparisons. After we ranked the genes according to the Bayesian ODP statistic, we employed the Bayesian FDR to control multiple test errors. The posterior probability of a gene being non-DE can be interpreted as a local FDR [16]. A direct estimator of FDR [17] can be computed based on the posterior probability *z*_*i*_. Specifically, the posterior expected FDR is

$$\begin{array}{lll} \bar{FDR}=E(FDR|data) & = & E\left(\frac{\sum \delta_{i}(1-r_{i})}{D}|data\right)\\ & = & \frac{\sum \delta_{i}(1-z_{i})}{D}, \end{array}$$

where *D *is the number of total rejections, indicator *δ*_*i *_= 1 if the *i*th gene is identified as a hit (its Bayesian ODP statistic ranks among the top *D*), and *δ*_*i *_= 0 otherwise. Plugging in the posterior probability *z*_*i*_, we obtained an estimated FDR. Controlling the Bayesian FDR at 5%, we produced a list of 363 genes identified as hits.

Sixty eight genes from the list were retested using the same reagent (Dhar-macon siRNA) as in the original experiment, all of which turned out to be positive, showing a remarkably high level of reproducibility. Through empirical testing, the gamma tubulin ring complex (*γ*TURC) is known to modulate paclitaxel sensitivity in a broad variety of non-small cell lung cancer cell lines. Thus selected genes from the complex can be considered landmark hits. The Bayesian ODP selected all the seven major components of the *γ*TURC (TUBGCP2, TUBA8, TUBGCP5, 76P, TUBGCP3, TUBG2, TUBG1). Considering the same number of selected genes (363), the original ODP produced 4 major components of the *γ*TURC (TUBG1, TUBA8, TUBG2, TUBGCP2), and the other five methods produced at most 5 of the major components.

Without knowing the list of truly DE genes, we could not compare the Bayesian ODP and other competing methods accurately based on the HTS lung cancer data. To overcome this problem, we used the Golden Spike data [18] to compare the Bayesian ODP with the other six methods included in the simulation study.

The Golden Spike dataset includes two conditions, with 3 replicates per condition. Each array has 14,010 probesets among which 3,866 probsets have spike-in RNAs. Among these 3,866 spike-in probsets, 2,535 probsets have equal concentrations of RNAs under the two conditions and 1,331 probsets are spiked in at different fold-change levels, ranging from 1.2 to 4-fold. Compared to other spike datasets, the Golden Spike dataset has a large number of probsets that are known to be DE, which makes it very popular for comparing differential expression methods.

There have been criticisms of the Golden Spike data set [19-21]. One of the undesirable characteristics is that the non-DE probesets have non-uniform p-value distributions. Irizarry *et al*. [20] identified a severe experimental artifact, which is that "the feature intensities for genes spiked-in to be at 1:1 ratios behave very differently from the features from non-spiked-in genes". Pearson [22] suggested that one can use the Golden Spike dataset as a valid benchmark with the 2,535 equal fold-change probsets as the true negatives instead of including the non-spiked-in probsets. As such, there are 1,331 true positives and 2,535 true negatives. Opgen-Rhein and Strimmer [4] proposed to remove the 2,535 equal fold-change probsets, leaving in total 11,475 genes, and 1,331 known DE genes. In this paper, we conducted the analysis in both cases, with the former denoted as Scenario 1 and the latter Scenario 2. We used the distribution free weighted method (DFW) [23] as the expression summary measure.

**Figure 7:**
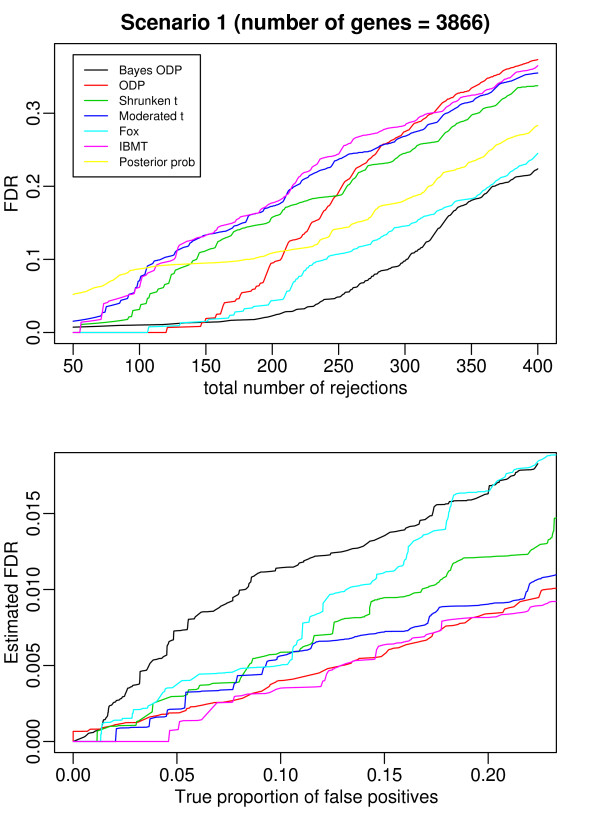
**Results from the comparison based on the Golden Spike dataset under Scenario 1 where the total number of genes to compare is 3866**. The top figure plots the FDR versus the total number of rejected genes. The bottom figure plots the estimated FDR versus the true proportion of false positives.

**Figure 8:**
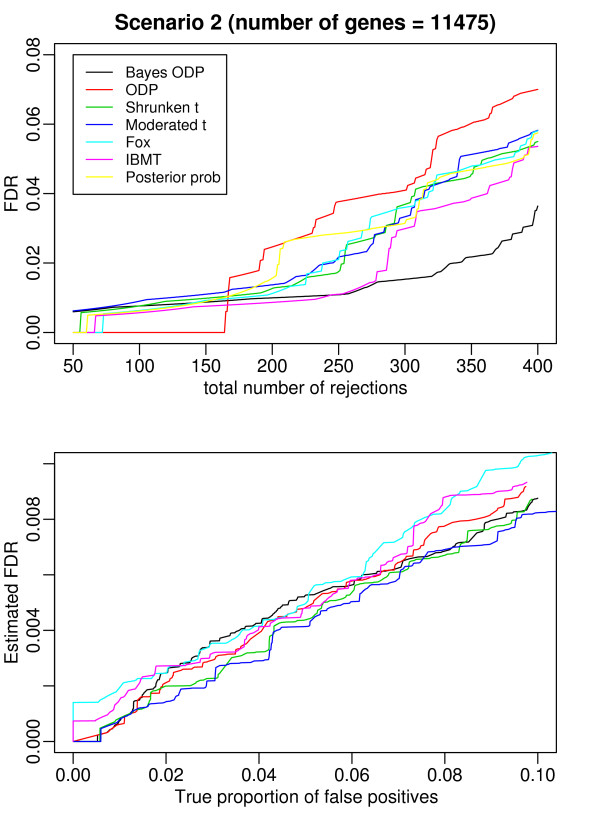
**Results from the comparison based on the Golden Spike dataset under Scenario 2 where the total number of genes to compare is 11475**. The top figure plots the FDR versus the total number of rejected genes. The bottom figure plots the estimated FDR versus the true proportion of false positives.

In addition to comparing the power of the seven methods given the same number of selected genes, we also compared their ability to correctly estimate the FDR. Because the null distributions of some of the test statistics (i.e., the Bayesian ODP, the original ODP, the shrunken *t*) are unknown, the Benjamini-Hochbergwe FDR procedure [24] can not be applied. We estimated the FDR by permutation analysis [3,13]. The upper panels of Figure 7 and Figure 8 plot the true FDR versus the number of selected genes under the two scenarios. In general, the Bayesian ODP outperforms the other methods in both scenarios. In Scenario 2, the Bayesian ODP has a 1% FDR when the total number of rejections is less than 160, while the original ODP has a zero FDR. Note that the difference is caused only by one gene that is a false negative. As the total number of rejections increases, the Bayesian ODP has a much smaller FDR than the original ODP. Fox and IBMT have the second best performance under Scenario 1 and Scenario 2, respectively. We provided the list of the first 400 genes, along with their true expression status, identified by the competing methods under each scenario in Additional file 2 and 3.

The lower panel of Figure 7 and Figure 8 compare the estimated FDR with the true proportion of false positives [9], from which we can assess the ability of the methods to correctly establish the statistical significance of DE genes. We did not include the posterior probability because its permutation-based FDR assessment is computationally intractable (it requires MCMC simulation on thousands of datasets, each generated by replacing a gene with a simulated null gene). All of methods in the comparison underestimate the number of false positives, which is consistent with the results reported in [9]. Correctly estimating FDR when the null distribution is unknown remains a challenge.

## Conclusion

One important feature of high throughput screening is that the number of replicates is extremely small, rarely exceeding 3. Full Bayesian hierarchical models were shown to be less competitive compared with some existing frequentist and empirical Bayes methods [9]. This is because full Bayesian models usually employ noninformative priors which do not provide suffcient shrinkage in the estimation. In this paper, we demonstrate that the full Bayesian model can be made a competitive approach by simply adding a point mass component in the variance prior. This modification introduces adequate shrinkage which improves the performance of the full Bayesian model considerably. The Bayesian computation is efficient. It takes about 10 minutes to run the FOR-TRAN program on a HP laptop (Pentium(R)4 CPU 3.20 GHz, 1 GB RAM) to analyze the lung cancer data.

The optimal discovery procedure (ODP) is one of the current developments in multiple testing. It has shown significant improvements over many leading methods. The full Bayesian model can be further combined with the ODP statistic. The Bayesian ODP can perform better than the original ODP, especially when there are few replicates in HTS. The Bayesian ODP employs the posterior probability of a gene being DE which naturally accounts for the uncertainty in the estimation of the null set. The parameter estimates in the original ODP, which are the sample mean and sample variance of individual test, are not reliable with few replicates. By replacing those with the estimates from the Bayesian model, the ODP is improved by a joint force of shrinkage estimation and borrowing strength across tests.

## Authors' contributions

JC, XX, and SZ developed the methods. JC and XX implemented and applied the methods. JC and SZ wrote the manuscript. AW and MAW provided the lung cancer HTS data and tested the analysis results.

## Supplementary Material

Additional file 1 **Bayesian ODP R code**. This file contains the R code to calculate the posterior probability from the Bayesian model and the Bayesian ODP. Click here for file

Additional file 2 **List of selected DE genes under Scenario 1**. This file contains the list of the DE genes, along with their true expression status, identified by the different methods from the Golden Spike dataset under Scenario 1 (excluding the 10144 non-spiked-in probsets). Click here for file

Additional file 3 **List of selected DE genes under Scenario 2**. This file contains the list of the DE genes, along with their true expression status, identified by the different methods from the Golden Spike dataset under Scenario 2 (excluding the 2,535 equal fold-change probsets). Click here for file

## References

[B1] Cui X, Churchill GA (2003). Statistical tests for differential expression in cdna microarray experiments. Genome Biology.

[B2] Tusher VG, Tibshirani R, Chu G (2001). Significance analysis of microar-rays applied to transcriptional responses to ionizing radiation. Proceedings of the National Academy of Sciences.

[B3] Cui X, Hwang JTG, Qiu J, Blades NJ, Churchill GA (2005). Improved statistical tests for differential gene expression by shrinking variance components estimates. Biostatistics.

[B4] Opgen-Rhein R, Strimmer K (2007). Accurate ranking of differentially expressed genes by a distribution-free shrinkage approach. Statistical Applications in Genetics and Molecular Biology.

[B5] Baldi P, Long AD (2001). Bayesian framework for the analysis of mi-croarray expression data: regularized t-test and statistical inference of gene changes. Bioinformatics.

[B6] Fox RJ, Dimmic MW (2006). A two-sample Bayesian t-test for microarray data. BMC Bioinformatics.

[B7] Lonnstedt I, Speed T (2002). Replicated microarray data. Statistica Sinica.

[B8] Smyth GK (2004). Linear models and empirical Bayes methods for assessing differential expression in microarray experiments. Stat Appl Genet Mol Biol.

[B9] Sartor MA, Tomlinson CR, Wesselkamper SC, Sivaganesan S, Leikauf GD, Medvedovic M (2006). Intensity-based hierarchical Bayes method improves testing for differentially expressed genes in microarray experiments. BMC Bioinformatics.

[B10] Kendziorski CM, Newton MA, Lan H, Gould MN (2003). On parametric empirical Bayes methods for comparing multiple groups using replicated gene expression profiles. Statistics in Medicine.

[B11] Lonnstedt I, Britton T (2005). Hierarchical Bayes models for cDNA mi-croarray gene expression. Biostatistics.

[B12] Storey JD (2007). The optimal discovery procedure: A new approach to simultaneous significance testing. Journal of the Royal Statistical Society, Series B.

[B13] Storey JD, Dai JY, Leek JT (2007). The optimal discovery procedure for large-scale significance testing, with applications to comparative microarray experiments. Biostatistics.

[B14] Storey JD (2002). A direct approach to false discovery rate. Journal of the Royal Statistical Society, Series B.

[B15] Whitehurst AW, Bodemann BO, Cardenas J, Ferguson D, Girard L, Pay-ton M, Minna JD, Michnoff C, Hao W, Roth MG, Xie X, White MA (2007). Synthetic lethal screen identification of chemosensitizer loci in cancer cells. Nature.

[B16] Efron B, Tibshirani R, Storey JD, Tusher V (2001). Empirical Bayes analysis of a microarray experiment. Journal of the American Statistical Association.

[B17] Newton MA, Noueiry A, Sarkar D, Ahlquist P (2004). Detecting differential gene expression with a semiparametric hierarchical mixture method. Biostatistics.

[B18] Choe SE, Boutros M, Michelson AM, Church GM, Halfon MS (2005). Preferred analysis methods for Affymetrix GeneChips revealed by a wholly defined control dataset. Genome Biology.

[B19] Dabney AR, Storey JD (2006). A reanalysis of a published Affymetrix GeneChip control dataset. Genome Biology.

[B20] Irizarry RA, Cope LM, Wu Z (2006). Feature-level exploration of a published Affymetrix GeneChip control dataset. Genome Biology.

[B21] Gaile DP, Miecznikowski JC (2007). Putative null distributions corresponding to tests of differential expression in the Golden Spike dataset are intensity dependent. BMC Genomics.

[B22] Pearson RD (2008). A comprehensive re-analysis of the Golden Spike data: Towards a benchmark for differential expression methods. BMC Bioinformatics.

[B23] Chen Z, McGee M, Liu Q, Scheuermann RH (2007). A distribution free summarization method for Affymetrix GeneChip arrays. Bioinformatics.

[B24] Benjamini Y, Hochberg Y (1995). Controlling the false discovery rate: a practical and powerful approach to multiple testing. Journal of the Royal Statistical Society B.

